# Therapeutic interventions for chronic central serous chorioretinopathy: a comprehensive assessment of systematic reviews

**DOI:** 10.1186/s40942-025-00660-x

**Published:** 2025-03-23

**Authors:** Henok Getahun, Rajendra S. Apte

**Affiliations:** John F. Hardesty, MD, Department of Ophthalmology and Visual Sciences, Washington University School of Medicine, St. Louis, MO 63110 USA

**Keywords:** Central serous chorioretinopathy, Photodynamic therapy, Verteporfin, Subthreshold micropulse therapy, Selective retina therapy

## Abstract

**Background:**

A variety of different treatments have been proposed to effectively treat chronic central serous chorioretinopathy but there remains uncertainty regarding the efficacy of a number of treatment options. We aim to evaluate the efficacy of several therapeutic options for chronic central serous chorioretinopathy including photodynamic therapy, conventional laser photocoagulation, subthreshold micropulse laser, selective retina therapy, vascular endothelial growth factor (VEGF) antagonists, and mineralocorticoid receptor antagonists.

**Methods:**

Pubmed, Embase, and Cochrane databases were searched for systematic reviews and meta-analyses evaluating treatment modalities for chronic central serous chorioretinopathy. Primary outcome measures included improvement in best corrected visual acuity (BCVA) and resolution of subretinal fluid (SRF). Conclusions regarding the efficacy of each modality were summarized and compared to findings of several key randomized controlled trials.

**Results:**

Ten systematic reviews and meta-analyses that incorporated 58 unique randomized controlled trials and observational studies were identified. Treatments that were shown to improve BCVA and promote SRF resolution included half-fluence and half-dose photodynamic therapy, conventional laser therapy, and subthreshold micropulse laser therapy. Evidence regarding selective retina therapy was limited and inconclusive. VEGF antagonists were not effective in the absence of choroidal neovascularization and mineralocorticoid receptor antagonists were not effective.

**Conclusion:**

The most effective therapeutic option for chronic central serous chorioretinopathy is half-dose or half-fluence photodynamic therapy, however, conventional laser therapy is an acceptable alternative in cases when photodynamic therapy is unavailable and when fluid leakage sites are not subfoveal or juxtafoveal. Subthreshold micropulse laser is less effective but can be considered when other options are unavailable.

**Supplementary Information:**

The online version contains supplementary material available at 10.1186/s40942-025-00660-x.

## Introduction

Central serous chorioretinopathy (CSC) is a pachychoroid disease characterized by choroidal vessel hyperpermeability and defects in the retinal pigment epithelium (RPE) [1]. These abnormalities may be due to impaired autoregulation of choroidal circulation, potentially related to glucocorticoid or sympathomimetic activity and result in increased choroidal vessel hydrostatic pressure and subsequent fluid leakage through the RPE [2, 3]. Hypercoagulability may also play a role in pathogenesis, with thrombi formation and elevated blood viscosity potentially affecting choroidal microcirculation and contributing to increased hydrostatic pressure [4]. However, the precise pathophysiologic mechanism of CSC needs further elucidation. Nonetheless, the anatomic sequelae of CSC are more clearly defined, with the resultant subretinal fluid (SRF) accumulation causing overlying serous retinal detachment and potential RPE atrophy [1]. Additionally, CSC can be complicated by choroidal neovascularization (CNV) that may compound losses in vision [5].

The clinical manifestations of CSC include reduced visual acuity and diminished contrast sensitivity with up to 13% of patients with persistent CSC qualifying as legally blind at 10 years after initial diagnosis [1, 3]. Additionally, CSC is a relatively common retinal disease with an incidence of 9.9 and 1.7 per 100,000 males and females, respectively [6].

Considering the substantial morbidity associated with CSC, it is important to have consistent guidelines for treatment based on sound clinical evidence. However, reaching such consensus has been difficult due to the unique challenges of studying CSC, including the nebulous classification system, the variable natural history, recurrent nature of the condition and the imprecise understanding of its pathogenesis. Multiple CSC subtypes have been proposed, with the most often cited delineation being between acute and chronic CSC (cCSC). There is a lack of consensus regarding the distinguishing characteristics of cCSC, with most definitions incorporating various durations of persistent serous retinal detachment while some studies incorporate cases of recurrent SRF accumulation [1, 7]. Notably, a classification system based on multimodal imaging findings has recently been proposed in which CSC cases are described as either simple or complex based on the total area of RPE alterations present, with further delineations based on history of SRF recurrence, resolution of SRF, duration of SRF persistence, presence of outer retinal atrophy or intraretinal fluid, presence of CNV, and presence of atypical features such as RPE tears [8]. However, due to a paucity of clinical trials strictly utilizing this classification system, providing treatment guidance based on the proposed definitions is difficult. By contrast, treatment guidelines based on more traditional definitions relying on serous detachment duration are currently easier to formulate, despite the lingering concern about the heterogeneity regarding classification.

In acute CSC, SRF may resolve spontaneously within 2–3 months. In most situations, observation, self-monitoring with an Amsler grid until follow-up and discontinuation of any factors that can trigger CSC such as corticosteroids is generally an acceptable treatment strategy [1]. However, cCSC may necessitate more active treatment to facilitate resolution [1]. To this end, a wide variety of treatment modalities have been proposed for cCSC, but the efficacy of several reported therapies is uncertain. We examined systematic reviews and meta-analyses assessing treatment options for cCSC and compared findings with key randomized controlled trials (RCTs) with the aim of summarizing and critically evaluating the existing evidence for proposed treatments.

## Methods

### Study eligibility criteria

Inclusion criteria for articles reviewed included full-text, open access systematic reviews and meta-analyses available in English from which results could be isolated to apply to author defined- cases of cCSC alone. Included studies also fulfilled prespecified criteria regarding the problem, intervention, and outcomes of interest (Table 1).

**Table 1:** PICO fields

Problem	Chronic central serous chorioretinopathy
Intervention	Half fluence photodynamic therapy, half dose photodynamic therapy, conventional laser therapy, subthreshold micropulse laser therapy, selective retina therapy, anti-VEGF agents, mineralocorticoid receptor antagonists
Comparison	Not applicable
Outcomes	Best corrected visual acuity improvement and subretinal fluid resolution

### Search strategy

Pubmed, Embase, and Cochrane Database of Systematic Reviews were searched for systematic reviews and meta-analyses assessing treatments for cCSC. We searched for articles that reviewed half dose photodynamic therapy (HD PDT) with Verteporfin, half fluence photodynamic therapy (HF PDT) with Verteporfin, conventional laser therapy, subthreshold micropulse laser therapy (SMLT), selective retina therapy (SRT) using microsecond laser pulses targeting the RPE, anti-VEGF agents, and/or mineralocorticoid receptor antagonists (MRAs). The database search concluded on October 18, 2024.

**Fig. 1:**
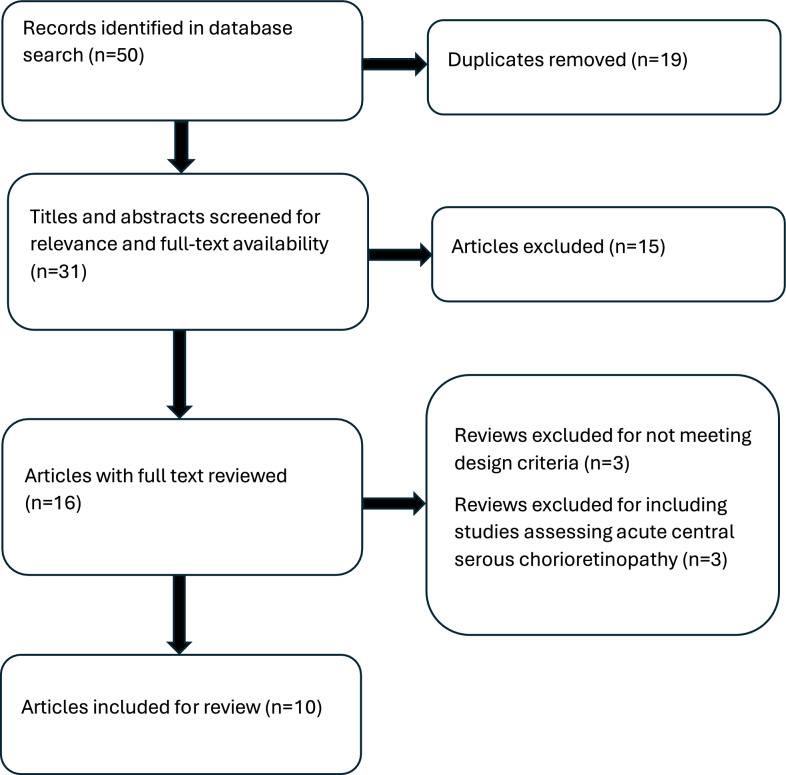
Review Selection Process. Flowchart depicting review selection process

Cochrane reviews were searched by narrowing the search field by topic to “retinal disease” and identifying articles assessing CSC. Pubmed and Embase were searched by filtering the study type to include only systematic reviews and meta-analyses. The search strategy for Pubmed and Embase included key word combinations for each treatment modality following the PICO format (Additional file 1). This expansive search incorporated articles that assessed HF PDT (25 J/cm^2^), HD PDT (3 mg/m^2^), conventional laser therapy, SMLT, SRT, anti-VEGF pharmacotherapy, and/or MRAs. The study selection process is summarized in Fig. 1.

### Data extraction and interpretation

The following data was extracted from included systematic reviews and meta-analyses: the name of the first author, title, publication year, inclusion and exclusion criteria, number and design of studies assessing therapies of interest incorporated in review, number of eyes receiving therapies of interest that were incorporated in review, publication year of studies assessing therapies of interest incorporated in review, narrative description of each review’s findings regarding the effect of therapies on best corrected visual acuity (BCVA) and SRF resolution, and when applicable, quantitative results from meta-analyses regarding effect of therapies of interest on BCVA and SRF resolution. Findings from systematic reviews and meta-analyses were compared to key RCTs regarding the efficacy of treatment options of interest.

### Study appraisal

A Measurement Tool to Assess Systematic Reviews (AMSTAR) 2 was used to critically appraise included systematic reviews and meta-analyses with scores ranging from critically low, low, moderate, to high [9].

## Results

### Included studies

Only one Cochrane review had been published regarding efficacy and safety of treatment modalities for cCSC at the time of the literature search [10]. Embase and Pubmed searches yielded nine relevant systematic reviews and meta-anlyses [11–19]. In total, the included systematic reviews and meta-analyses incorporated evidence from 58 unique randomized controlled trials and observational studies (Table 2). Of the relevant reviews identified, two were graded as having a high level of evidence, two with low level of evidence, and six with a critically low level of evidence according to the AMSTAR 2 tool (Table 3) [9].

**Table 2:** Evidence profile

Number of studies overall	58
Number of randomized control trials	20
Number of observational studies	38
Study years	2008–2021
Date of literature search	Up to October 18 2024
Number of eyes	2567

**Table 3:** Summary of evidence

Article	Comparator	Narrative description of BCVA Improvement (WMD or SMD; 95% CI; p value)	Narrative description of SRF resolution (OR or RR; 95% CI; p value)	Strength of evidence
HF PDT				
Photodynamic Therapy for Central Serous Chorioretinopathy (Erikitola et al.) [11]	N/A	No significant BCVA difference between HF PDT and anti-VEGF treatment in RCTs. HF PDT was effective at improving BCVA at 1,3, 6, and 12 months in observational studies	In RCTs, 75% of eyes treated with HF PDT had resolved SRF at 12 months compared to 25% for the anti-VEGF group	Critically low
System Review and Meta-Analysis on Photodynamic Therapy in Central Serous Chorioretinopathy (Ma et al.) [12]	FF PDT	No difference between HF PDT and FF PDT in improving BCVA at 1 month, 3 months, and 12 months after treatment	No statistically significant difference in SRF resolution between HF PDT and FF PDT	Critically low
HD PDT				
Photodynamic Therapy for Central Serous Chorioretinopathy (Erikitola et al.) [11]	N/A	Observational studies showed improvement in BCVA at 1, 3, 6, and 12 months after treatment	Majority of studies included in review reported at least a 75% resolution rate of SRF by final follow up	Critically low
Comparative Efficacy of Treatment for Chronic Central Serous Chorioretinopathy: A Systematic Review with Network Meta-Analyses (van Dijk et al.) [13]^a^	N/A	PDT (included HD PDT and HF PDT) had a significant effect on BCVA improvement at 2 months after treatment (− 0.13 logMAR; 95% CI: − 0.20 to − 0.06 logMAR; p = 0.00021)	PDT showed significant efficacy in inducing SRF resolution at 2 months after treatment (20.6; 6.3 to 66.7; p < 0.0001)	Low
Conventional Laser Therapy				
Comparative Efficacy of Treatments for Chronic Central Serous Chorioretinopathy: A Systematic Review with Network Meta-Analyses (van Dijk et al.) [13]	N/A	Conventional laser was effective at improving BCVA at 2 months post treatment (− 0.17 logMAR; − 0.31 to − 0.03 logMAR; p = 0.019)	Conventional laser was effective at inducing resolution of SRF at 2 months after treatment (36.4; 2 to 655.7; p = 0.015)	Low
Subthreshold laser therapies^b^				
Interventions for central serous chorioretinopathy: A Network Meta-Analysis (Salehi et al.) [10]	Sham laser treatment	SMLT showed superior BCVA improvement at 6 months after treatment compared to sham laser (-0.38 logMAR; -0.56 to -0.20 logMAR)	N/A	High
Comparing Interventions for Chronic Central Serous Chorioretinopathy: A Network Meta-analysis (You et al.) [14]^c^	Control and HD PDT	Compared to control, subthreshold laser was effective at improving BCVA at 3 months after treatment (− 0.11 logMAR; − 0.20 to − 0.02 logMAR; p = 0.017). Compared to PDT, there was no significant advantage of one treatment over the other (− 0.11 logMAR; − 0.25 to 0.026 logMAR)	Compared to control, subthreshold laser was effective at inducing SRF resolution at 3 months after treatment (2.45; 1.23 to 4.88; p = 0.011). Compared to PDT, there was not a significant difference (1.49; 0.58 to 3.81)	Low
Comparative Efficacy of Treatment for Chronic Central Serous Chorioretinopathy: A Systematic Review with Network Meta-Analyses (van Dijk et al.) [13]	Control	SMLT was effective at improving BCVA at 2 months after treatment compared to non-treatment (− 0.16 logMAR; − 0.29 to − 0.03 logMAR; p = 0.013). SRT did not have a statistically significant effect on BCVA improvement at 2 months compared to control (− 0.03 logMAR; − 0.09 to 0.03; p = 0.27)	No statistically significant effect of SMLT inducing SRF resolution at 2 months follow up, compared to control (13.5; 0.9 to 207.6; p = 0.062). SRT did have a statistically significant improvement in SRF resolution compared to control (3.4; 1.7 to 6.8; p = 0.00075)	Low
Comparison of the Efficacy and Safety of Subthreshold Micropulse Laser with Photodynamic Therapy for the Treatment of Chronic Central Serous Chorioretinopathy (Wu et al.) [15]	HD PDT	SMLT was more effective at improving BCVA at 6 months after treatment (− 0.15 logMAR; − 0.23 to -0.07 logMAR; p < 0.01)	SMLT was not significantly different from HD PDT in SRF resolution after over 6 months after treatment (0.661; 0.414 to 1.055; p = 0.107)	Critically low
Anti-VEGF agents				
Interventions for central serous chorioretinopathy: A Network Meta-Analysis (Salehi et al.) [10]	HF PDT	No significant BCVA improvement at 12 months after treatment (0.03 logMAR; − 0.08 to 0.15 logMAR)	N/A	High
Comparative Efficacy of Treatment for Chronic Central Serous Chorioretinopathy: A Systematic Review with Network Meta-Analyses (van Dijk et al.) [13]	N/A	No significant BCVA improvement at 2 months after treatment (0.02 logMAR; − 0.12 to 0.15 logMAR; p = 0.82)	No significant effect at inducing SRF resolution (0.8; 0.4 to 2.4; p = 0.83)	Low
*Intravitreal Anti-Vascular Endothelial Growth Factor for the Treatment of Chronic Central Serous Retinopathy: a Meta-Analysis of the Literature (Palakkamanil *et al*.)* [16]	N/A	Weighted mean BCVA did not differ significantly before and after treatment with anti-VEGF agents	Weighted overall percentage of SRF resolution was 68.4% with no comparator in pooled analysis	Critically low
MRAs				
Comparing Interventions for Chronic Central Serous Chorioretinopathy: A Network Meta-analysis (You et al.) [14]	Placebo	No significant improvement of BCVA at 3-month follow-up (− 0.023 logMAR; − 0.07 to 0.024 logMAR)	No significant effect at inducing SRF resolution at 3 months follow-up (1.46; 0.23 to 9.35)	Low
Comparative Efficacy of Treatment for Chronic Central Serous Chorioretinopathy: A Systematic Review with Network Meta-Analyses (van Dijk et al.) [13]	N/A	No significant effect on improving BCVA at 2 months follow-up (− 0.05 logMAR; − 0.10 to 0.01 logMAR; p = 0.099)	No significant effect on inducing SRF resolution at 2 months follow-up (1; 0.4 to 2.4; p = 0.99)	Low
Mineralocorticoid Receptor Antagonists in Chronic Central Serous Chorioretinopathy (Sanhueza and Gonzalez) [17]	N/A	MRAs had a small effect on BCVA improvement compared to control (− 0.06 logMAR; margin of error: − 0.1 to − 0.02 logMAR)	No significant effect on SRF height (MD: -83.6, margin of error: -178.7 to 11.6; N/A)	Critically low
Mineralocorticoid Receptor Antagonists for Chronic Central Serous Chorioretinopathy: Systematic Review and Meta-Analysis (Felipe et al.) [18]	Control	Effect of MRAs on BCVA ranged from clinically unimportant improvement to clinically unimportant worsening (0.22 logMAR, 95% CI: − 0.04 to 0.48 logMAR)	No significant effect on reducing SRF height (SMD: -0.35; 0.95 to 0.26)	High
Efficacy and Safety of the Mineralocorticoid Receptor Antagonist Treatment for Central Serous Chorioretinopathy: a Systematic Review and Meta-Analysis (Duan et al.) [19]	Control	No significant improvement of BCVA at 1 month (− 0.02 logMAR; − 0.06 to 0.03 logMAR) and 3 months (0 logMAR, − 0.06 to 0.06 logMAR) after treatment	N/A	Critically low

*SMD* standard mean difference, *WMD*, weighted mean difference, *MD* mean difference, *CI* confidence interval, *OR* odds ratio, *RR* relative risk, *N/A* not applicable, *RCT* randomized controlled trial, *logMAR* logarithm of minimum angle of resolution, *BCVA* best corrected visual acuity, *SRF* subretinal fluid, *PDT* photodynamic therapy, *HF PDT* half fluence PDT, *FF PDT* full fluence and full dose PDT, *HD PDT* half dose PDT, *SMLT* subthreshold micropulse laser therapy, *SRT* selective retina therapy, *anti-VEGF* anti-vascular endothelial growth factor, *MRA* mineralocorticoid receptor antagonist

^a^van Dijk et al. incorporated HD PDT and HF PDT into one group for meta-analysis

^b^Section termed subthreshold laser therapies includes reviews evaluating SMLT and/or SRT

^c^You et al. grouped SMLT and SRT into one group for meta-analysis

### HF and HD PDT

Three systematic reviews evaluating HF PDT or HD PDT were identified and all reported moderate efficacy in improving BCVA and achieving SRF resolution up to 12 months after treatment [11–13]. Erikitola et al. incorporated 2 RCTs evaluating HF PDT and 13 observational studies evaluating either HF PDT or HD PDT [11]. All studies included in this review reported improved BCVA at final follow-up, with all but one study incorporated in the review reporting statistically significant improvements. Additionally, all studies included in the review reported SRF resolution in at least 75% of cases of cCSC at 12 months after treatment with HF PDT or HD PDT. Ma et al. conducted a meta-analysis including studies evaluating treatments for both acute and chronic CSC [12]. The only intervention arm that could be isolated to cCSC involved the comparison of HF PDT and full fluence PDT (FF PDT), which incorporated data from two observational studies. At each follow-up time assessed, there was no statistically significant difference in BCVA improvement or SRF resolution between the two treatment protocols. Notably however, choroidal regional nonperfusion at three months follow-up was more common after FF PDT (Odds ratio: 0.09, 95% CI: 0.03 to 0.29, p < 0.001), indicating that HF PDT may have a superior safety profile. Finally, a meta-analysis conducted by van Dijk et al. included six RCTs and evaluated HF PDT and HD PDT as a single group [13]. Results from this review yielded significant improvements in BCVA and higher odds of SRF resolution at two months after treatment compared to non-treatment.

### Conventional laser therapy

One meta-analysis evaluated conventional laser therapy and noted its efficacy in improving BCVA and resolving SRF at two months after treatment, although only one RCT assessing this treatment was included [12]. Furthermore, participants who received conventional laser therapy in this study did not have subfoveal or juxtafoveal fluid leakage, reflecting the current practice pattern of avoiding conventional laser in such cases due to risk of inducing central or paracentral scotoma [20]. By contrast, an earlier prospective RCT of argon laser photocoagulation demonstrated earlier resolution of SRF without any benefit on visual acuity [21].

### Subthreshold micropulse laser therapy

Four systematic reviews evaluated SMLT efficacy in cCSC. Notably, the reviews included studies with various laser settings. For example, Salehi et al. incorporated studies evaluating 810nm micropulse diode laser whereas You et al. grouped studies assessing 810nm micropulse diode laser and 527nm near infrared laser into a single arm termed subthreshold laser therapy [10, 14]. In the review by Salehi and colleagues, results yielded significant improvement in BCVA at six months follow-up after treatment with SMLT, however only one RCT assessing this treatment was included [10, 22]. You et al. conducted a meta-analysis that evaluated subthreshold laser therapies utilizing data from two RCTs evaluating near infrared laser and micropulse diode laser therapy [14]. Results of the meta-analysis suggested an improvement in BCVA and a resolution of SRF at three months after treatment compared to control groups. Interestingly, pairwise comparison between the subthreshold laser therapy group and a PDT group showed no statistical differences in the same outcome measures at three months, despite the PDT group achieving higher overall rates of SRF resolution. Additionally, a meta-analysis conducted by van Dijk et al. reported a statistically significant effect of SMLT treatment on BCVA improvement at two months after treatment, and a positive, yet statistically insignificant effect on SRF resolution [13]. Finally, the meta-analysis conducted by Wu et al. yielded results supporting SMLT efficacy, reporting more positive effects in BCVA and SRF outcomes at six months follow up than HD PDT in pairwise comparison [15]. Of note, findings from the key PLACE trial support the superior efficacy of HD PDT compared to SMLT [23].

### Selective retina therapy

SRT alone, rather than grouped with SMLT, was assessed in one meta-analysis [13]. This review incorporated three RCTs evaluating SRT, all with the similar laser settings (Q-switched neodymium-doped yttrium lithium fluoride laser, wavelength 527 nm, pulse duration 1.7 μs, spot diameter 200 μm, pulse repetition rate 100 Hz) [13, 24–26]. Results from the meta-analysis yielded mixed results, with a statistically significant improvement in SRF resolution at two months follow-up compared to control, but without a significant effect on BCVA at the same follow-up time.

### Anti-VEGF agents

Three meta-analyses evaluated anti-VEGF agents, primarily utilizing studies assessing ranibizumab or bevacizumab [10, 13, 16]. None of the meta-analyses yielded significant results with respect to BCVA improvement or SRF resolution. Notably, all the reviews incorporated studies that defined the presence of CNV as an exclusion criterion. However, results of the MINERVA trial do support the use of anti-VEGF agents for CSC cases complicated by CNV [27].

### Mineralocorticoid receptor antagonists

MRAs such as eplerenone and spironolactone were evaluated in five of the included reviews, all supporting the conclusion that MRAs did not have a clinically impactful effect on BCVA or SRF resolution [13, 14, 17–19]. Side effects from MRA treatment were rare but included gynecomastia, infection, hyperkalemia, intermittent dizziness, and fatigue [13, 19]. Results of the reviews that were included are in accordance with findings from the VICI and SPECTRA trials, which highlight the lack of efficacy of MRAs and the superior efficacy of HD PDT over MRAs, respectively [28, 29].

## Discussion

Systematic reviews, meta-analyses, and several clinical trials support the practice of using HD PDT or HF PDT as a primary treatment modality for cCSC with the acceptability of conventional laser therapy in cases without subfoveal or juxtafoveal leakage and when HF PDT and HD PDT are unavailable. Findings from Ma et al. supported the clinical practice of using HF PDT or HD PDT rather than FF PDT due to potential for fewer adverse effects [12]. The most common side effects of PDT with verteporfin include injection site reactions and visual disturbances such as blurred vision, however other adverse reactions such as infusion-related back pain and hypersensitivity reactions are also possible [30]. HF PDT and HD PDT have particularly favorable safety profiles with low rates of visual disturbances, choroidal hypoperfusion, and RPE atrophy [31–33]. Therefore, we posit that the initial treatment strategy for CSC cases with SRF persistence of at least three months should be either HF PDT or HD PDT. Although indications for retreatment were not evaluated in included systematic reviews, available evidence suggests that retreatment may be considered if there is persistent SRF or fluid leakage on indocyanine green angiography after three-month follow-up [1, 31–34]. Although Wu et al. reported greater efficacy of SMLT over HD PDT with regard to some outcome measures, both observational and RCT studies were incorporated into meta-analysis, introducing potential bias [15]. There were limited definitive results regarding this pairwise comparison from other reviews included, with You et al. finding no statistically significant difference between PDT and SMLT with respect to BCVA improvement and SRF resolution at three months follow-up [14]. Although the majority of the reviews reported some treatment benefit of SMLT, results were more mixed than the evidence regarding HD PDT and HF PDT [13]. Taken together with the findings of the recent PLACE trial, we believe it is reasonable to regard HD PDT and HF PDT as more efficacious at the present time [23]. SMLT may be a reasonable alternative when PDT is unavailable and conventional laser is not warranted. The cumulative evidence regarding SRT was considerably more limited and supported anatomic benefits of treatment without significant effect on functional outcomes. SRT may be a promising alternative in the future, however, further studies are warranted to make definitive statements regarding treatment efficacy. By contrast, anti-VEGF agents should not be used in cases without secondary CNV and MRAs appear to lack efficacy in treating cCSC.

There are several limitations to our review. Several of the included systematic reviews and meta-analyses incorporated observational studies which may have considerable bias and variation. An additional source of potential bias is the high variability in defining cCSC in the included reviews, with some authors relying on definitions from individual studies that were incorporated and others utilizing predefined criteria of serous detachment lasting longer than three months. Furthermore, reviews evaluating SMLT incorporated numerous studies with various laser settings such as 577 nm and 810 nm SMLT. Finally, the majority of reviews included received a low or critically low score on appraisal based on the AMSTAR 2 tool. Notably, all the reviews that assessed HD PDT and HF PDT received either low or critically low scores. Although it is prudent to weigh reviews appropriately based on defined appraisal tools, it should be noted that potentially high-quality systematic reviews in other fields may have received low scores based on the AMSTAR 2 tool [35].

Given the potential of cCSC to result in permanent vision loss, the high-cost and current shortage of verteporfin, and the limited applicability of conventional laser therapy, identifying other efficacious treatments is an important undertaking [36]. Adequately powered RCTs are needed to assess experimental therapies. Perhaps even more crucial is the need for a universal classification system for CSC to better standardize cohorts recruited to future trials. An attempt should be made by authors of future trials to define CSC cases based on the classification system proposed by Chhablani and colleagues.

## Conclusion

Based on the numerous systematic reviews and meta-analyses included in this review, the primary treatments for cCSC should rely upon HD PDT or HF PDT. Additionally, conventional laser therapy is a reasonable alternative, especially in cases where the focal leakage is extrafoveal. In scenarios in which PDT is unavailable and the case is not amenable to conventional laser therapy, SMLT may be a reasonable alternative. Anti-VEGF agents should only be utilized in cases with secondary CNV. Meanwhile, MRAs should not be considered a reasonable treatment strategy at the current time. Finally, the usefulness of SRT for cCSC requires further study.

## Supplementary Information

Additional file 1. Search Strategy. Depicts the search strategy utilized to search Pubmed and Embase databases for systematic reviews and meta-analyses.

## Data Availability

No datasets were generated or analysed during the current study.

## Abbreviations

CSC: Central serous chorioretinopathy
RPE: Retinal pigment epithelium
SRF: Subretinal fluid
CNV: Choroidal neovascularization
cCSC: Chronic central serous chorioretinopathy
VEGF: Vascular endothelial growth factor
cCSC: Chronic central serous chorioretinopathy
RCT: Randomized controlled trial
PDT: Photodynamic therapy
HD PDT: Half dose photodynamic therapy
HF PDT: Half fluence photodynamic therapy
SMLT: Subthreshold micropulse laser therapy
SRT: Selective retina therapy
MRA: Mineralocorticoid receptor antagonist
PICO: Problem, interventions, comparisons, outcomes
BCVA: Best corrected visual acuity
AMSTAR: A measurement tool to assess systematic reviews
FF PDT: Full fluence PDT
CI: Confidence interval
logMAR: Logarithm of the minimum angle of resolution
