# Supplementary material for: Therapeutic interventions for chronic central serous chorioretinopathy: a comprehensive assessment of systematic reviews
Source: Int J Retina Vitreous. 2025 Mar 23;11:34. doi: 10.1186/s40942-025-00660-x (PMC11931883; doi:10.1186/s40942-025-00660-x)
Supplement: Supplementary file 1 — Additional file 1. Search Strategy. Depicts the search strategy utilized to search Pubmed and Embase databases for systematic reviews and meta-analyses. [file 40942_2025_660_MOESM1_ESM.pdf]

Search strategy:

(("central serous chorioretinopathy" OR "central serous chorio-retinopathy" OR "CSC") AND ("photodynamic therapy" OR "PDT" OR "half fluence" OR "half-fluence" OR "half-dose" OR "half dose" OR "laser therapy" OR "subthreshold laser" OR "micropulse laser" OR "SMLT" OR "subthreshold micropulse" OR "selective retina therapy" OR "SRT" OR "mineralocorticoid antagonist" OR "MRA" OR "spironolactone" OR "eplerenone" OR "anti-VEGF" OR "VEGF-antagonist" OR "VEGF antagonist" OR "vascular endothelial growth factor antagonist" OR "ranibizumab" OR "bevacizumab") AND ("efficacy" OR "effectiveness" OR "vision" OR "improvement" OR "BCVA" OR "subretinal fluid" OR "SRF")) Filters: Meta-Analysis, Systematic Review
